# Growing Up Together in Society (GUTS): A team science effort to predict societal trajectories in adolescence and young adulthood

**DOI:** 10.1016/j.dcn.2024.101403

**Published:** 2024-06-06

**Authors:** Eveline A. Crone, Thijs Bol, Barbara R. Braams, Mark de Rooij, Barbara Franke, Ingmar Franken, Valeria Gazzola, Berna Güroğlu, Hilde Huizenga, Hilleke Hulshoff Pol, Loes Keijsers, Christian Keysers, Lydia Krabbendam, Lucres Jansen, Arne Popma, Gert Stulp, Nienke van Atteveldt, Anna van Duijvenvoorde, René Veenstra

**Affiliations:** a Erasmus School of Social and Behavioral Sciences, Erasmus University Rotterdam, the Netherlands; b Leiden University, Institute of Psychology, the Netherlands; c Department of Sociology, University of Amsterdam, the Netherlands; d Department of Clinical, Neuro, and Developmental Psychology, Faculty of Behavioral and Movement Sciences, Vrije Universiteit Amsterdam, the Netherlands; e Radboud University Medical Center, Donders Institute for Brain, Cognition and Behaviour, Departments of Cognitive Neuroscience and Human Genetics, Nijmegen, the Netherlands; f Social Brain Lab, Netherlands Institute for Neuroscience (KNAW) and University of Amsterdam, Amsterdam, the Netherlands; g Department of Psychology, University of Amsterdam, the Netherlands; h Department of Experimental Psychology, Utrecht University, the Netherlands; i Department of Child and Adolescent Psychiatry & Psychosocial Care, AmsterdamUMC and Research Institute Amsterdam Public Health, Amsterdam, the Netherlands; j University of Groningen, Department of Sociology / Inter-University Center for Social Science Theory and Methodology, Groningen, the Netherlands

## Abstract

Our society faces a great diversity of opportunities for youth. The 10-year Growing Up Together in Society (GUTS) program has the long-term goal to understand which combination of measures best predict societal trajectories, such as school success, mental health, well-being, and developing a sense of belonging in society. Our leading hypothesis is that self-regulation is key to how adolescents successfully navigate the demands of contemporary society. We aim to test these questions using socio-economic, questionnaire (including experience sampling methods), behavioral, brain (fMRI, sMRI, EEG), hormonal, and genetic measures in four large cohorts including adolescents and young adults. Two cohorts are designed as test and replication cohorts to test the developmental trajectory of self-regulation, including adolescents of different socioeconomic status thereby bridging individual, family, and societal perspectives. The third cohort consists of an entire social network to examine how neural and self-regulatory development influences and is influenced by whom adolescents and young adults choose to interact with. The fourth cohort includes youth with early signs of antisocial and delinquent behavior to understand patterns of societal development in individuals at the extreme ends of self-regulation and societal participation, and examines pathways into and out of delinquency. We will complement the newly collected cohorts with data from existing large-scale population-based and case-control cohorts. The study is embedded in a transdisciplinary approach that engages stakeholders throughout the design stage, with a strong focus on citizen science and youth participation in study design, data collection, and interpretation of results, to ensure optimal translation to youth in society.

## 1 Introduction to Growing Up Together in Society (GUTS)

Societal contribution is defined as the capacity to contribute to goals for self (well-being and mental health) and other individuals or groups (contributions to others) (Fuligni, 2019). Adolescence and young adulthood, jointly defined as the period between the ages of 10 and 24 years (Sawyer et al., 2018), are important periods for the development of societal contributions; they mark the transition period from childhood, characterized by a strong dependence on parents and caregivers, to adulthood, when one is expected to function as a mature, independent individual (e.g., politically, economically, and socially) and to commit to the social norms of society (Dahl et al., 2018). Contributing to goals for self and others can occur in various societal domains; these include educational achievements, such as investing in the future and staying committed to school success (Blair and Raver, 2015), and social contributions, such as cooperation, sharing, and helping others, while refraining from antisocial behaviors and balancing personal wellbeing (Veenstra and Laninga-Wijnen, 2021). How adolescents grow up individually cannot be disentangled from the social and societal network in which they grow up, reinforcing the intertwined contribution of individual development, social development, and societal influences (Choudhury et al., 2023).

Becoming a contributing citizen, including feeling needed and useful (Fuligni et al., 2022), emerges from the complex interplay between nature and nurture, where our genetic makeup interacts with internal (e.g., hormone changes) and external (e.g., social experiences) environmental factors that shape brain development and the ability to adapt and thrive in society. Structural Magnetic Resonance Imaging (sMRI) studies have shown that there are continuous changes in brain structure throughout adolescence and into early adulthood. These changes are observed in cortical regions, the evolutionarily younger areas of the brain important to focus on goals against distractors and obstacles (Tamnes et al., 2017), and in subcortical regions, the evolutionarily older areas showing greater inter-individual variation in developmental trajectories and important for processing motivational and affective signals (Wierenga et al., 2018). The impact of individual genes on brain development varies across the lifespan, facilitating changes in e.g. the brain’s neurotransmission and hormonal systems, sleep regulation, and behavior (Brouwer et al., 2022, Gao et al., 2019, van Soelen et al., 2012). A large-scale study including five twin cohorts across the lifespan (N=861, ages 9–70-years) yielded heritability estimates of brain structure change ranging from 16 % in subcortical regions to 42 % in cortical regions, demonstrating a significant effect of genetic makeup on brain structure change (Brouwer et al., 2017). Heritability estimates were higher in adults than in children, suggesting a larger influence of environmental factors on brain development in childhood and adolescence compared to adulthood (Brouwer et al., 2017, Van der Meulen et al., 2020).

Although our knowledge of biological inter-individual differences has improved, a pressing issue concerns the need to relate our understanding of individual brain developmental trajectories to the major transitions that occur in how individuals successfully pursue personal and societal goals (Choudhury et al., 2023). Inequalities in family opportunities and support affect how youth can benefit from education and ultimately their changes to contribute to academic and social outcomes (Andrews et al., 2021). Particularly in understanding the transitions that take place across adolescence and emerging adulthood, it is important to move beyond the study of individuals and examine individuals in the context of diverse social and societal opportunities. These include their family context (Smetana and Rote, 2019), educational settings (Blair and Raver, 2015), social connections both offline and online (such as increased communication through social media) (Armstrong-Carter and Telzer, 2021), and diverse societal contexts (Palacios-Barrios and Hanson, 2019) and demands (Hails et al., 2019, Raver, 2004). Diversity in demands can range from growing up in disadvantaged or affluent environments to navigating the complexities when growing up in challenging circumstances (Blair and Raver, 2016, Choudhury et al., 2023).

The Growing Up Together in Society (GUTS) program seeks to break new ground by examining the societal contributions of youth using a novel theoretical framework combining individual (biological, behavioral), social and societal perspectives. The novel framework of the GUTS program posits that the development of adaptive self-regulation in diverse social and societal contexts is a key factor in explaining why some adolescents and young adults are more successful than others in navigating societal and social challenges (Wesarg-Menzel et al., 2023). Not all young people have similar opportunities to contribute to society, and not all individuals are equally capable of making contributions (Fuligni, 2019), especially in periods that are marked by large societal challenges, such as the COVID-19 pandemic (Masten and Motti-Stefanidi, 2020). Inter-individual differences in the ability to self-regulate may protect against or accelerate (i.e., moderate) potential detrimental effects of unequal opportunities on personal and societal outcomes (Moffitt et al., 2011). Inter-individual differences in self-regulatory abilities may also directly explain (i.e., mediate) the relation between diverse social opportunities and individual contribution to society at multiple levels (Hails et al., 2019).

Several studies have examined longitudinal trajectories in separate domains, including brain development (Brouwer et al., 2022, Tamnes et al., 2017, Teeuw et al., 2018), social networks (Gremmen et al., 2017), and antisocial behavior (Moffitt, 2018), but very few have integrated these perspectives into a single study design. As insights often emerge at the intersection of scientific disciplines (Park et al., 2023), the GUTS program aims to include diversity across a variety of societal contexts (Dotson and Duarte, 2020) and combine the study of these different domains. The major goal of the GUTS program is to understand why some young people thrive in making positive contributions to the needs of others, while others have difficulty placing societal goals above personal goals and engage in, for example, delinquent behavior (Moffitt, 2018). The subgoals of the GUTS program are to examine the models of self-regulation in diverse contexts of neurobiological and socio-economic opportunities, academic context, social networks and antisocial behavior, using one overarching research design.

In subsequent parts of this article, we outline the reasoning behind the team science effort of the GUTS program. We first unpack the psychological and neural processes of self-regulation, a key skill for navigating societal contexts that is expected to be relevant to quantifying, predicting, and explaining pathways to societal contributions (Robson et al., 2020). Next, we describe two prominent models in developmental science that suggest that self-regulation may moderate (i.e., influence) or mediate (i.e., explain) the relation between diversity of biological and societal opportunities and societal contributions. Later, we explain the importance of including diversity in social/societal context as a research goal in social developmental neuroscience studies. We provide the details and metadata of the GUTS program in Box I. Finally, we describe how predictive modeling can advance theory development (Rosenberg et al., 2018) and how participatory action research can advance the validity of study design (Choudhury et al., 2023, van Atteveldt et al., 2019). In the conclusion section, we describe the benefits of this approach for future research programs.

## 2 Behavioral and neural pathways of self-regulation

Contributing to society involves multiple processes that depend on the balance between short- and long-term goals, as well as goals related to outcomes for oneself and others (Fuligni and Galvan, 2022). This balance ultimately leads to individual well-being, educational attainment, social connections, and positive impact on others (Wesarg-Menzel et al., 2023). Individual characteristics can contribute to the development of socially and civically adaptive citizens. We propose that self-regulation when navigating multiple contexts is a key process that influences and/or explains how individuals, with different opportunities, become engaged contributors to society (Hofmann et al., 2012, Robson et al., 2020) who feel needed and useful (Fuligni et al., 2022).

We define self-regulation as the process of deliberate control over behavior when balancing immediate and future-oriented goals, and balancing self-oriented and other-oriented goals (Wesarg-Menzel et al., 2023). Self-regulatory abilities, which are at the core of successful social adjustment, include three components: goal setting, goal motivation, and goal capacity (Hofmann et al., 2012, Wesarg-Menzel et al., 2023). Goal setting involves the pursuit of higher-order individual goals, and the development of how individuals use values to guide this process occurs during adolescence (Wesarg-Menzel et al., 2023). Adolescents choose their goals in an increasingly independent manner, and these goals have increasingly far-reaching consequences. From a societal perspective, these goals may be long-term, such as investing in education, supporting others, or short-term, such as seeking sensation, sometimes at the expense of others (for example when striving for social status). Goal motivation refers to the ‘drive’ or effort expended in pursuit of the goal, which is influenced by sensitivity to different types of rewards (e.g., peer approval or monetary gains) (Eccles and Wigfield, 2002, Wesarg-Menzel et al., 2023) and motivational self-beliefs (Burnette et al., 2013). Goal capacity refers to the ability to keep the goal in mind, monitor progress, and inhibit distractions; it requires key neurocognitive functions such as working memory, inhibition, and error monitoring (Nigg, 2017). Goal capacity also includes goal flexibility: the ability to switch between goals, especially between goals that contradict or even undermine each other. This is particularly important during adolescence, when the expectations and demands of parents, caregivers, and teachers do not necessarily align with adolescents’ goals, and when there is a transition from parental monitoring to self-regulation (Farley and Kim-Spoon, 2014, Lionetti et al., 2019, Zeman et al., 2006). The transition to young adulthood requires expanding current goals to include social and occupational goals, taking into account increasing financial responsibilities, future-oriented career goals, and supportive social networks (Massey et al., 2008, Wesarg-Menzel et al., 2023).

From a neuroscience perspective, the neurodevelopment of self-regulation has been studied using a variety of paradigms in combination with functional Magnetic Resonance Imaging (fMRI). These studies showed that cortical areas are important for the setting of higher order goals (Whitaker et al., 2018). These goals are cognitively constructed, often intentionally pursued, and can motivate behavior in pursuit of that goal (Davidow et al., 2018, Geier et al., 2010, Luna et al., 2015). Prior studies showed increased recruitment of frontal and parietal cortical brain regions in the use of goal capacity during adolescence (Casey, 2015). Specifically, the ability to inhibit contextually inappropriate behavior increases from childhood to adulthood, along with increased activity in frontal, temporal-parietal, striatal, and thalamic areas (Casey, 2015, Crone and Steinbeis, 2017, Luna et al., 2015). An interesting, yet largely unanswered question is the role of genetic make-up in the development of self-regulation. It is clear that brain structural and functional traits all have a heritable component, including those relevant for self-regulation (e.g. Medland et al., 2022), and also most of the cognitive and behavioral traits linked to the development of self-regulation (as well as disorders involving problems with self-regulation) are heritable (for a review, see Hulshoff-Pol et al., this issue). Yet, few studies have aimed to estimate effect sizes of genetics in studies of self-regulation and/or delineate the molecular (genetic) mechanisms underlying self-regulation. With the advent of genome-wide association studies (GWAS) and increased data sharing mentality in the scientific community, we now have tools in hand to explore the molecular mechanisms and design variables that allow us to estimate the genetic contribution to self-regulation (e.g. in the form of polygenic scores (Allegrini et al., 2022). First examples of such studies include GWAS of delay discounting (Sanchez-Roige et al., 2018) and risk tolerance (Karlsson Linner et al., 2019; for a more detailed review of this subject, see Hulshoff-Pol et al., this issue).

The drive to pursue these goals is influenced by sensitivity to affective and motivational signals (Casey, 2015, van Duijvenvoorde et al., 2016). Neuroscience has identified a potential indicator of variation in sensitivity to goal motivation. A meta-analysis of over 100 studies including a wide variety of reward tasks validated a neural marker of reward sensitivity consisting of a network of the subcortical ventral striatum and prefrontal cortex (Liu et al., 2011). Meta-analyses in adolescent populations have shown an increase in activity in these reward-related brain centers during mid- to late adolescence, correlating with hormonal changes associated with pubertal development (Braams et al., 2015, Silverman et al., 2015). These changes have been most extensively studied in the context of monetary gains, but have also been demonstrated for social stimuli such as facial expressions (Guyer et al., 2008, Pfeifer et al., 2011), social inclusion (Chein et al., 2011), and social acceptance (Achterberg et al., 2016, Guyer et al., 2012). The motivational drive for potential rewards coincides with a flexible recruitment of frontal-parietal cortex (also referred to as goal capacity regions) and frontal-temporal (temporal-parietal and superior temporal) cortex (also referred to as social brain regions), creating a window of social-affective responsivity and goal flexibility (Blakemore and Robbins, 2012, Crone and Dahl, 2012, Dahl et al., 2018). These enhanced motivational levels, along with slowly developing self-regulation and social perspective-taking, can make adolescents more susceptible to risky decisions such as increased alcohol consumption, engaging in dangerous driving, and delinquent behavior (Casey et al., 2011, Goldstein and Volkow, 2002). These same motivational signals may also make adolescents more susceptible to prosocial choices, resulting in increased concern for social relationships and helping others (Telzer, 2016, Telzer et al., 2014). Thus, while these signals can provide motivation, or a ‘drive’, they can also pose challenges in the presence of motivational conflicts, such as those between immediate and long-term benefits or between self- and other-related benefits (Crone and Fuligni, 2020, Kotabe and Hofmann, 2015, Luna et al., 2015, Telzer, 2016).

Although developmental transitions have been described for different forms of self-regulation separately, including goal setting, motivation, capacity, and flexibility (Tervo-Clemmens et al., 2023), the relation between self-regulation and societal trajectories can best be measured in experimental tasks that combine different aspects of self-regulation (Nigg, 2017). An important paradigm that examines the balancing of goals for the self over time is the delay discounting task (Luerssen et al., 2015). In this task, participants can choose between an immediate smaller reward or a delayed larger reward. Choosing the delayed reward decreases when the reward is further in the future or when the immediate reward is larger, a process known as delay discounting which requires future orientation (Ikink et al., 2023, van den Bos et al., 2015). Various studies have shown that the ability to delay gratification increases between childhood and adulthood, which researchers have interpreted as a developmental increase in goal capacity (Peper et al., 2018, Steinberg et al., 2008). Yet, delay of gratification is also dependent on whether adolescents grow up in an environment that is uncertain or harsh, where delaying gratification is not always the optimal strategy (Fenneman and Frankenhuis, 2020), therefore the most adaptive goal capacity and setting strategies are contextually dependent (Wesarg-Menzel et al., 2023).

We recently developed a social delay discounting task as a central measure in the GUTS program, extending the paradigm developed in prior research (Albrecht et al., 2011), in which participants are asked to make delay decisions not only for themselves, but also for friends and strangers, thereby varying goal setting from close to distant partners (Van Rijn et al., 2024). Whereas previous research showed that goal motivation (reward orientation) and capacity (delay of gratification) were associated with maturation of brain regions important for balancing reward processes and cognitive control (van den Bos et al., 2015), our recent research shows that goal setting for friends and strangers relative to self was associated with activity in brain networks important for social perspective taking, specifically the medial prefrontal cortex, precuneus and temporal-parietal junction (TPJ) (van de Groep et al., 2023, Van Rijn et al., 2024). Together, these findings suggest that the neural signatures of social self-regulation as measured using the social delay of gratification task can contribute to our understanding of the development of goal setting, goal motivation and goal capacity and the inter-individual differences in (including contextual influences on) these trajectories.

## 3 Self-regulation as a pathway to societal contribution

**Fig. 1:**
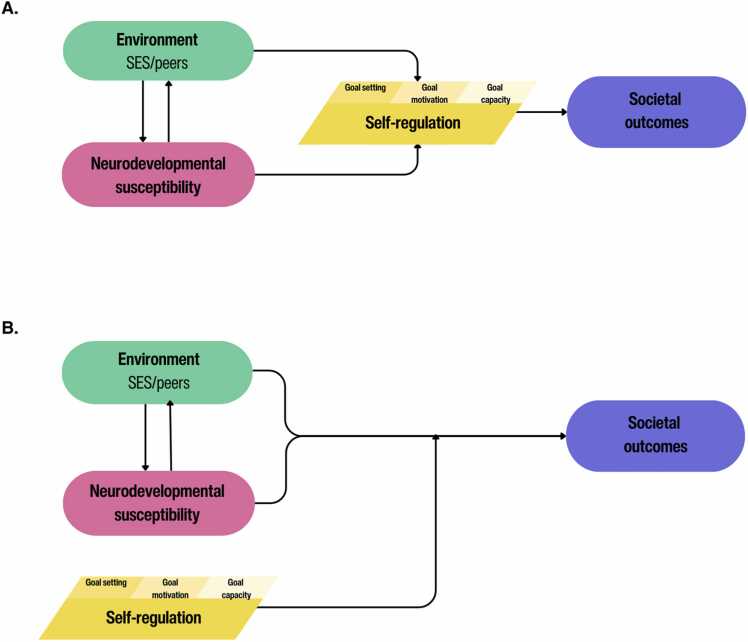
The GUTS program aims to test mediation (A) and moderation (B) models for self-regulation (goal setting, goal motivation, goal capacity) to test the relations over time between neurobiological sensitivities, social/societal context (SES, peer network, neighborhood influences) and societal outcomes. Societal outcomes include academic outcomes, social outcomes, societal engagement outcomes, mental health/well-being outcomes, and outcomes developed in co-creation with youth.

We conceptualize adolescence as a period of significant biological and environmentally induced changes in self-regulatory abilities, defined as an increased drive to set one’s own goals, heightened sensitivity to personal and social rewards, and maturing goal capacity (Wesarg-Menzel et al., 2023). Self-regulation should be interpreted as an umbrella term for the range of processes that are captured under goal setting, goal motivation and goal setting, including the ability to understand goals (i.e., the cognitive capacity to understand societal values) as well as the affective feeling that are associated with certain goals (i.e., the feeling and caring about needs of others). In our theoretical framework, we aim to quantify two prominent models of influence hypothesized in developmental science, self-regulation as a mediator, and self-regulation as a moderator of the relation between biological and societal opportunities and outcomes (see Fig. 1). Specifically, we expect that the development of self-regulation will underlie (i.e., mediate) and/or influence (i.e., moderate) the relation between societal opportunities and academic and social outcomes (Hails et al., 2019, Healy et al., 2021, Pollak et al., 2020, Robson et al., 2020). Biological and environmental changes can influence adolescent behavior at many levels, such as motivations to develop autonomy within the family and do well in school, to fit into social groups, or to deviate from expected societal norms and engage in antisocial behavior (Barnes et al., 2022, Brieant et al., 2020, Kim-Spoon et al., 2017, Li et al., 2019). Biological and environmental changes have been examined separately in different fields of research, resulting in a lack of comprehensive understanding of what motivates youth to contribute to individual goals in different societal contexts (Choudhury et al., 2023). We propose in the GUTS program that genetic, hormonal, and brain development are biological opportunities (Brouwer et al., 2022) and that societal experiences (social-economic status, parental support) are social/societal opportunities (Green et al., 2023, Keijsers et al., 2022) which together predict future contribution to society. We hypothesize that the development of balanced self-regulatory abilities (goal setting, goal motivation, goal capacity/flexibility) will explain and/or influence the relation between diverse biological and social/societal opportunities and individual contributions to society at academic and social levels (Wesarg-Menzel et al., 2023). These relations can explain societal contributions through various pathways. For example, wanting to feel appreciated by a community group may make it more likely that adolescents regard the benefits of this group as more personally relevant (goal setting). Caring about the peer group and feeling connected, could be an intrinsic motivation to contribute to the group (goal motivation). Acting on these motivations because you expect to be appreciated and valued for this contribution can promote the capacity to contribute (goal capacity). We propose that functional brain development (such as cortical brain changes important for setting adaptive goals and subcortical brain changes associated with increased reward sensitivity in individual adolescents) provide potential indicators of self-regulation (goal setting, goal motivation, goal capacity) in adolescence (van Duijvenvoorde et al., 2016).

Given the protracted developmental trajectory of self-regulation, both mean levels and slopes of development are expected to shape future outcomes (Crone and Elzinga, 2015). Those who begin with high levels of self-regulation, or who develop more rapidly toward them, are expected to have more advantageous outcomes. If self-regulation is influenced by social/societal opportunities (including socioeconomic resources, social networks, antisocial experiences), and in turn influences personal, social and societal outcomes, then self-regulation is expected to be one of the underlying processes of the relation between social opportunities and outcomes. According to this mediation model, brain functioning and the development of self-regulation are shaped and influenced by education, family, and social networks, which in turn shapes contributions to society (in terms of investing in the well-being, education, and social needs of other groups by helping, sharing, and supporting others to achieve a goal) (Brieant et al., 2020). According to the moderation model, inter-individual differences in the ability to self-regulate may buffer or accelerate the potentially detrimental effects of opportunity inequality on personal and societal outcomes (Kim-Spoon et al., 2017).

In terms of societal contribution, research has been inconclusive in understanding the relations between domains of contribution (e.g. academic, social, societal), in part because much research effort has been devoted to understanding transitions from the perspective of a single research domain, focusing on main effects of predictors rather than on sets of interacting predictors from different domains. Whereas the ability to delay gratification may be a critical predictor in, for example, the academic domain, self-regulation in relation to others may be more important in the social network domain. However, this distinction only becomes apparent when various contexts are included in one research program. Therefore, within the GUTS program we distinguish between five domains of contribution: academic contribution (school/work success), social contribution (quality of social relations), societal contribution (political, contribution to welfare of outgroups) and contribution to self (mental health, well-being). We allow for a fifth domain which is developed in co-creation with adolescents (Green et al., 2023).

The longitudinal approach in the GUTS program will be key to its success. Prior research suggests that self-regulation influences both the contributions individuals can make to society and enables individuals to become the architect of the social environment that will influence their ability to self-regulate (Brieant et al., 2020). Disentangling these bidirectional influences requires the ability to track processes over time. In Box 1 we describe how we aim to achieve these goals in the GUTS program.

Box 1 **GUTS program design and meta-data access.**

**Fig. 2:**
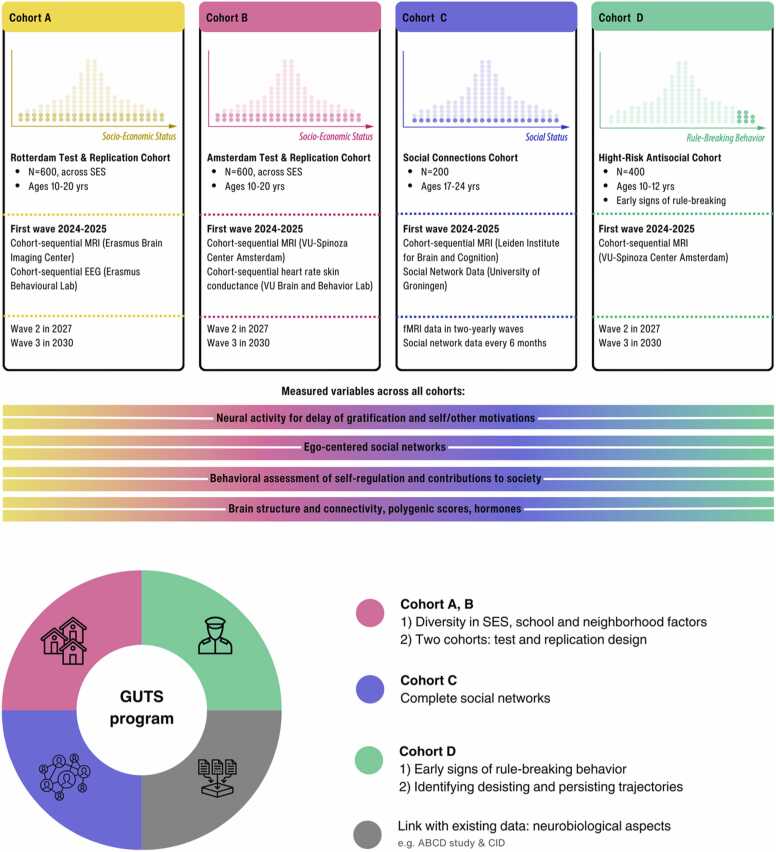
The GUTS program aims to comprehensively test and integrate the development and contextual influence on self-regulation and societal outcomes, across multiple work packages in a 10-year program. Top: Display of cohorts and overlapping constructs. The dots represent a schematic presentation of the population from which we aim to sample. For cohorts A and B we sample from the whole range of Socio-Economic Status (SES), for Cohort C we sample from the whole range of social status withing a sociometric network and for Cohort D we sample specifically from the population of youth that shows rule breaking behavior. Bottom: A schematic overview of the overarching goals. See Table 1 for an overview of measures that are collected across all work packages and Box 1 for a description of the GUTS program.

To understand how differences in social and societal contexts influence how societally engaged young people become, the GUTS program consists of three work packages dedicated to the collection of multimethod longitudinal cohort data and a fourth work package to builds upon existing data. A notable strength of this design is its distinctive ability to assess self-regulation, and outcome measures across domains, individuals, ages, and time. Fig. 2A shows the anticipated design across four cohorts

- Cohort A/B: A test and replication cohort. In work package academic/social development: We aim to enroll 1200 participants aged 10–20 years of representative samples, across a range of full socio-economic status divided across 2 cohorts. The sequential design where participants are included at different starting ages is advantageous because it efficiently captures developmental trajectories over a 10-year period of time.

- Cohort C: In work package social networks: We aim to include 400 participants aged 18–20 years, for whom the whole social network will be included.

- Cohort D: In work package antisocial development: We aim to enroll 400 participants aged 10–12 years who have a criminal record.

The first measurement is planned in 2024–2025. Participants will be followed up in measurement waves in 2027–2028 (when participants are 13–23 years old) and 2030–2031 (when participants are 16–26 years old). New participants in the same age group will be recruited in case of attrition.

Table 1 describes full metadata for the measures that are acquired across all cohorts. Each wave will include a laboratory visit including several cognitive and behavioral tasks and questionnaires and saliva harvesting for DNA extraction outside the scanner as well as several MRI measurements including:

o Indices of functional brain development using fMRI and the Social Delay Discounting Task for self and other (Van Rijn et al., 2024)

o Resting-state fMRI to map functional connectivity independent of a specific task.

o Structural brain scans to quantify differences and changes in brain architecture including cortical and subcortical gray matter volumes.

Individual work packages will include additional fMRI tasks to quantify differences in the neural correlates of trust, reward sensitivity, empathy, and prosocial behavior. For a subsample of participants, EEG will be included to examine the fast temporal dynamics of reward processing and self-regulation in different social contexts. In subsamples, Experience Sampling Methods (ESM) will be included to understand how self-regulation functions in everyday life and real-time settings (Myin-Germeys and Kuppens, 2022).

The GUTS program will use both existing and newly validated measures. The overall goal of the program is to examine the combined societal, social, behavioral, and biological mechanisms that drive the transitions from adolescence to emerging adulthood, and the impact of these transitions on how young people function in educational settings, social relationships, and society. The full study design and the hypotheses for each subproject can be found on OSF page: https://osf.io/wntx4

All GUTS procedures will be communicated transparently throughout the study, and data will be stored according to the FAIR principles, organized according to the BIDS standard (Gorgolewski et al., 2016); see also the GUTS RDM handbook that facilitates data and metadata harmonization for more details: https://guts-consortium.github.io/guts-rdm/). This will allow us to make the data available for future studies worldwide. For these purposes, as well as for internal data sharing, the GUTS data management group is building a system based on iRODS (open-source data management software; https://irods.org/) and Yoda (Smeele et al., 2024) that maximizes intuitive data findability and accessibility while preserving personal data privacy. Structured datasets are stored in iRODS/Yoda, where metadata can be extracted automatically, thus separating the personal data from their descriptive metadata. Harmonized metadata are then made available publicly via an external, user-facing metadata explorer that provides researchers with a user-friendly data filtering and “basket checkout” functionality data for tailored dataset access requests. Access requests can be handled by data managers, and after approval by a data access committee the data subset will be automatically available on a specified Yoda instance that is available to authorized requesters only.

Follow the GUTS program on: https://www.gutsproject.com

**Table 1:** Overview of measures that are collected across all work packages within the GUTS program. See Supplementary Information for the references.

**Category**	**Construct**	**Measurement**
**Predictor variables**		
*Demographic Background*	Demographics	Age, cultural background, ethnicity
	Demographics	Biological/birth sex, perceived sex
	Physical health	Physical health and medication use
	SES	Family background (highest completed education, perceived social status)^1^
	Neighbourhood Postal Code	Postal Code
	Neighbourhood violence	Neighbourhood Violence Scale (NVS) Youth^2^
	Perceived inequality	Perceived inequality^3^
	Romantic relationships	Romantic relationships, sexual orientation
	Cognitive Functions/IQ estimation	Raven^4^
	Puberty	Puberty Development Scale^5^
	Sleep	Chronic Sleep Reduction questionnaire
*Parenting and Family Support*	Family warmth, conflict	Network Relationship Inventory (subscales warmth, conflict)^6^
	Autonomy support	Perception of Parents Scale (subscale autonomy support)^7^
	Adverse Life Events	Adverse childhood events^8^
**Self-regulation**		
*Goal Setting*	Future goals	Aspiration Index (AI; shortened)^9^
*Goal Motivation*	Behavioural inhibition / approach systems	Behavioural Inhibition / Activation Scale (BIS/BAS)^10^
*Goal Capacity*	Self-control	Brief self-control scale^11^
	Perspective Taking and Empathy	Interpersonal Reactivity Index (IRI; subscales: empathic concern, perspective taking)^12^
*fMRI task*	Self-regulation for Self and Others	Social Delay Discounting Task^13^
**Biological variables**		
*Genetics*	DNA Extraction	Saliva
*Hormones*	Testosterone, cortisol and DHEA	Saliva
*Brain structure*	Structural Brain measures	High-resolution structural scan
*Brain connectivity*	Functional Brain Connectivity	Resting state scan
**Social networks**		
*Social networks*	Ego-centred social networks	Name generator & Composition
*Social Closeness*	Friendship closeness	Inclusion of Others in the Self (IOS) scale^14^
**Outcome variables**		
*Educational achievement*	Education Level	Highest completed/current educational level
	Educational aspirations	Idealistic educational aspirations
	Educational aspirations	Realistic educational aspirations
	Educational aspirations	Parental educational aspirations
*Social Relations*	Friendship quality	Network of Relationships Scale (NRI-SPV) short form^6^
	Social media use	Compulsive internet use^15^
	Online Prosocial Behaviour	Online Prosocial Behaviour Scale – Extended (OPBS-E)^16^
*Risk-taking behaviours*	General Risk taking	German Socio-Economic Panel (SOEP) – general/domain^17^
	Positive Risk Taking	Positive Risk-Taking scale^18^
	Smoking/alcohol	Basic set smoking/alcohol/marihuana/drugs
	Substance use	Alcohol Use Disorders Identification Test (AUDIT) ^19^
*Societal Engagement*	Civic Engagement	Contribution to Society Scale^20^
	Altruism	Altruism (PBQ-NL)^21^
*Societal perspectives*	Ethnocentrism	Ethnocentrism^22^
	Political orientation	Interdisciplinary Perspectives on Politics of Adolescents & Democracy Scale (IP-PAD)^23^
	Institutional Trust	Trust in institutions^24^
*Mental health/Wellbeing*	Mental health	Strengths and Difficulties Questionnaire (SDQ)^25^
	Quality of life	Youth Quality of Life – Short Form instrument (YQOL-SF)^26^
	Wellbeing	Multidimensional Wellbeing in Youth Scale (MWYS) – subscale self-confidence^27^
	Psychopathic traits	Youth Psychopathic Traits (YPI)^28^
	Perceived stress	Perceived Stress Scale^29^

## 4 Novel direction 1: Bringing diversity in socio-economic background as a research goal to the field of social neuroscience

Understanding the impact of diversity in socioeconomic circumstances in relation to inter-individual differences in neurobiological profiles is an important challenge we face as we seek to understand how young people make transitions from adolescence to adulthood across multiple domains (Dotson and Duarte, 2020). Previous studies have shown that children and adolescents who grow up in socioeconomically disadvantaged circumstances have more mental health problems (Reiss, 2013), more difficulties with self-regulation (Brieant et al., 2020, Brieant et al., 2023), different patterns of brain development (Buckley et al., 2019, Sheridan, 2023), and lower levels of (or opportunities for) civic engagement (Lenzi et al., 2012). Growing awareness of the detrimental effects of socioeconomic disadvantage on mental health, academic, and societal outcomes is making scientists more aware of the lack of diversity in previous research (Dotson and Duarte, 2020, Green et al., 2022). New directions in research are now increasingly incorporating diversity in SES as an important factor in explaining individual developmental trajectories (Brieant et al., 2023) and in generalizing our findings to the full range of youth growing up in today’s society (Fakkel et al., 2020).

Given that some children grow up in more challenging environments than others, for example, because their families have fewer socioeconomic resources (Andrews et al., 2021, Lareau, 2011), their peer networks are less supportive (Laursen and Veenstra, 2021), or their neighborhoods are less resourceful or more antisocial (Moffitt, 2018), an important question is to understand who finds their way in society despite disadvantageous environmental circumstances (Fenneman and Frankenhuis, 2020) and which adolescents have difficulty following societal rules and regulations, and engage in for example delinquent behavior (Moffitt, 2018). Our prior work on early offenders shows that some adolescents persist in antisocial behavior whereas others desist antisocial behavior, where desisting trajectories were associated with increased activity in the prefrontal cortex when regulating aggressive responses (van de Groep et al., 2022). These findings suggest that antisocial trajectories result from a combination of environmental circumstances and neurobiological sensitivities (Oostermeijer et al., 2016) that together influence self-regulation and societal outcomes.

An important question for future research is whether the development of self-regulation can buffer the effects of lower SES and/or neurobiological sensitivities on personal and societal outcomes, which would suggest that self-regulation training may be a powerful intervention to cope with the societal system-level disadvantages (self-regulation as a moderator). It may also be that lower SES influences the development of self-regulation, for examples because it is less advantageous to wait for a larger, delayed reward in the context of fewer resources, which in turn influences mental health and academic and societal outcomes (self-regulation as a mediator). Finally, given that self-regulation is a multi-dimensional construct, some aspects of self-regulation may be buffers, whereas other aspects may be influenced by SES. It should be noted that in both models, system-level interventions that reduce societal inequalities are the most desirable intervention for improving mental health and academic outcomes. In summary, if the goal is to bring more societal context to developmental neuroscience, SES is an important developmental context to consider for understanding intra and inter-individual differences (Dotson and Duarte, 2020, Taylor et al., 2020).

## 5 Novel direction 2: bringing social networks as a research goal to the field of social neuroscience

The transition to adolescence is characterized by profound changes in young people’s social relationships. The social network that surrounds young people can provide challenges (e.g., negative peer pressure) but also opportunities (e.g., modeling prosocial behavior) (Veenstra and Laninga-Wijnen, 2021). How adolescents grow up individually cannot be separated from the social network in which they grow up (Armstrong-Carter and Telzer, 2021, Güroğlu and Veenstra, 2021).

The amount of time adolescents spend with their peers increases substantially during adolescent development, and peer relationships become a significant developmental context that strongly influences their choices, motivation, and behavior (Lam et al., 2014). Parents remain important in the lives of adolescents, but they are becoming less hierarchical and allow their children more autonomy (Smetana and Rote, 2019). As adolescents move into emerging adulthood, parental regulation decreases, and peers’ self-regulation becomes a predictor of adolescents’ self-regulation (Farley and Kim-Spoon, 2014). In contrast, adverse peer experiences, such as victimization and rejection, can negatively influence self-regulation (Herd and Kim-Spoon, 2021). Throughout both adolescence and emerging adulthood, maintaining positive relationships within the family and forming and maintaining positive relationships with peers, such as friendships and romantic relationships, are prominent developmental tasks (Farley and Kim-Spoon, 2014, Herd and Kim-Spoon, 2021).

Adolescents and emerging young adults’ orientation and motivation to gain acceptance and status among their peers drive many goal-directed behaviors, but at the same time they must deal with the demands, expectations, and goals set by their parents, teachers, and our complex society. Young people have to make important decisions about their educational future early in life (as early as age 11–12 years in the Netherlands). Emerging adults face additional challenges in thinking about their future. They are expected to succeed in their education and careers, and eventually to become independent adults who balance their well-being with their contribution to society. Achieving this requires balancing short- and long-term goals, such as enjoying time with friends versus investing in education, and managing personal aspirations alongside societal goals, such as seeking short-term social rewards from friends (e.g. going to a party) versus the more delayed goals of making a lasting contribution to society.

Some of society’s expectations and demands conflict with adolescents and young adults’ short-term goals (e.g., seeking the thrill of intense sensations and going to parties), creating tension that can challenge self-regulatory processes and increase disconnection from family and peers. While society demands that adolescents become independent and build their futures as “good citizens”, society and social media often promote the pursuit of popularity and dominance, thus influencing the goals people seek (e.g., status at the expense of belonging) (Prinstein, 2017). To understand how self-regulation develops during adolescence and emerging adulthood, it is important to examine how self-regulatory processes and inter-individual differences in social reward sensitivity interact with these complex social dynamics. Given adolescents’ sensitivity to peer influence, this developmental period may represent a critical turning point for young people with poorer self-regulatory abilities, who may be particularly sensitive to immediate gratification and less focused on long-term perspectives for self and others, with implications for well-being and fewer opportunities to contribute to society (Laursen and Veenstra, 2021).

Young adulthood is also a time when self-regulation skills are further developed, and peers are influential. First, peers influence the goals that adolescents choose to pursue (Wesarg-Menzel et al., 2023). Most importantly, adolescents’ peer orientation is characterized by an increased need and concern to achieve peer status, which becomes a central goal in their daily lives. Second, adolescents’ motivation to achieve certain goals and engage in certain behaviors can be strongly influenced by behaviors that are approved or sanctioned by peers. Research has shown that in some contexts the mere presence of peers, especially high-status peers, can alter adolescents’ motivation to engage in risk-taking, antisocial, and prosocial behaviors (Powers et al., 2022, Somerville et al., 2019). Importantly, young adults are not passively exposed to their peer influence but can actively select whom to spend time with. Selecting peers with similar goals and avoiding peers whom they can predict to have an adverse influence on their goals can become a strategy to scaffold self-regulation, yet we still know very little about the neural mechanisms through which different young adults select their peers, and how the peers they select influences their neural activity (Parkinson et al., 2018). By tracking neural activity and behavior in relevant tasks over multiple years in all members of tightly knit social networks of young adults together with their academic and social achievements and position in the social network over several years, we expect to shed light onto the complex interaction between peer selection, peer influence and neural activity to better understand dynamics that benefit vs harm young adults (Güroğlu and Veenstra, 2021).

Box 2 **Fast Forward.**

Today’s society is highly complex, with rapidly evolving technologies and economic uncertainties, as evidenced by the recent pandemic and the urgent need to develop new ways of living sustainably. Youth in today’s society are growing up with social inequality, pressure to perform, and uncertainty. At the same time, each new generation of youth is driven by curiosity, reinventing possibilities and demonstrating resilience and creativity to address societal challenges that transcend the structures invented by adults. Within the GUTS program, our vision is defined by the urgency to fast-forward our perspectives on the needs of today’s youth to ensure a thriving future in a rapidly changing society, now and throughout the duration of the program. For example, past generations valued having a steady job to provide for the family, but today’s society embraces adaptability, mobility, and job flexibility where offline and online worlds are connected. In addition, much of youth innovation and social change stems from youth’s proclivities to challenge societal norms, suggesting that definitions of societal contribution may also change over time. Therefore, a longitudinal program such as GUTS needs to regularly reflect on whether our design captures the mechanisms of adolescent development that allow youth to grow, learn socially, and adapt in a rapidly changing world, and includes youth perspectives as experts on their own lives.

The GUTS program is novel in its approach: We use transdisciplinary youth participatory research in combination with psychological, neurocognitive developmental research, sociological research on family and policy-related inequalities, and individualized mental health research. This approach has the potential to produce interdisciplinary breakthroughs, practical solutions, and scalable interventions to improve the social position of youth. In our research, the involvement of societal stakeholders - such as youth panels, parents, young professionals, and policymakers - is a key element, as is our investment in developing practical insights and tools that are directly relevant to them. This approach is well established in the technical sciences, but not yet in the social sciences. A shared value and motivation of our research team members is that we are highly invested in youth, hearing voices from diverse perspectives, and creating positive societal change. As such, this program has the potential not only to understand mechanisms, but also to inform policy, practice, and education.

The GUTS team is a diverse and tightly knit team of psychologists, neuroscientists, sociologists, and family researchers who aim to examine how the developmental processes of self-regulation and social reorientation lead to well-being and contribution to society, with a particular focus on social inequalities in background and development. The consortium shares the mission to elucidate and predict the factors that contribute to the development of key competencies in growing up in modern complex societies. These include: a culture of open dialogue not only about science but also about the work environment/culture; a culture in which making mistakes is part of development; keeping an eye on the social atmosphere and potential indicators that the work culture requires attention; intervening quickly when a problem arises. Psychological safety and balancing work pressure are key to creating and maintaining our positive and creative work culture. We support our team members in times of life stress, such as caring responsibilities. Therefore, we personally embrace the ‘Fast Forward’ culture of youth by creating teams that are fresh, novel, driven, fast-moving, and motivated by shared academic values.

## 6 Steps for integration: theory building in the context of machine learning

Theory building can be advanced by recent methods that use machine learning algorithms to combine many different characteristics to predict outcomes (Molina and Garip, 2019). Since the turn of the century, there has been a shift in focus from explanatory modeling to predictive modeling (Breiman, 2001, Shmueli, 2010). This shift has led to major innovations in fields as diverse as data science, natural language processing, and biochemistry, and is beginning to find its way into social and neuro-sciences (Genon et al., 2022, Rosenberg et al., 2018, Yarkoni and Westfall, 2017). In machine learning, the term “prediction” specifically refers to out-of-sample prediction, which means the prediction of outcomes that were not present in the data used to train the model. This differs from traditional regression analysis strategies that focus on in-sample prediction. Often, in-sample prediction is a poor proxy for the more important out-of-sample prediction because of overfitting-- the model picking up on peculiarities in the data that do not generalize to novel cases (Yarkoni and Westfall, 2017). Out-of-sample predictive ability is a robust, objective measure that researchers can use to test the strength of their theories and models, and a useful quantity in determining how close a theoretical model is to practice.

Combining explanatory modeling with a focus on prediction allows researchers in the GUTS program to establish causal effects and properly quantify the importance of these effects in terms of how well we predict novel cases (Hofman et al., 2021). As such, predictive modeling is used as a way to make accurate predictions and as a way to check the robustness of the findings, similar to approaches used in psychological science or neuroscience (Woo et al., 2017, Yarkoni and Westfall, 2017).

One of the advantages of recent advances in predictive modeling is that such methods are better able to incorporate data from different domains and time scales than traditional models (Wu et al., 2023). This is important for the GUTS program because the data come from different domains, including brain measures, genetic information, social relationships, and behavioral measures to explain the emergence of contributions to society. However, simply concatenating all the data may not be an optimal strategy because it does not take into account the different properties of the domains. That is to say, the degree of measurement error will vary across domains (e.g., self-reports, hormones, fMRI signals), whereas others remain comparatively clean; therefore, it is important to take into account differences in reliability among the measures. To address these variations, we aim to develop a novel machine learning method capable of integrating different data domains with their distinct properties in terms of measurement error and timescales. This method will allow us to compare the predictive capabilities of these domains while evaluating the effectiveness of different theories. The advantage of these models is that a large number of variables across many different domains can together predict the likelihood of developmental outcomes. Thus, this program will move beyond single variables to a combined set of interacting variables in predicting such outcomes.

Within predictive modelling, out-of-sample predictive ability is often determined by training the model on one part of the data (e.g., 70 % of the data) and assessing the strength of the model on the remaining “out-of-sample” data. A major strength of the GUTS program is that data on multiple samples will be collected, meaning that models can first be trained on one sample and subsequently assessed and confirmed in a full novel sample. This is an asset for both explanatory and predictive modelling approaches that lead to robust, generalizable findings.

## 7 A fundamental research program with societal implications

There are societal and ethical consequences to the creation and publication of predictive models. It is important that the meaning and consequences of these predictions are well understood and communicated. For example, when scientists study predictive modeling, they study whether predictions become better than chance (or better than before). In contrast, when scientific findings are communicated to society, the word prediction is often understood in a more absolute way, in the sense that the results of a predictive model can accurately predict someone’s status. In addition, statistical predictions are not deterministic. Therefore, results and implications should be clearly communicated to avoid stigmatization and negative attitudes or expectations (Singh and Rose, 2009).

The GUTS program (Box I) will rely on a Responsible Research and Innovation approach to maximize benefits for individuals and society, and to anticipate and manage potential societal impacts. Responsible Research and Innovation is a governance framework that optimizes the alignment of the values and purposes of research with the values, needs and interests of society (Owen et al., 2012, van Atteveldt et al., 2019). There is a need for continuous reflection and dialogue with stakeholders, as the ambiguity and uncertainty of science and methodological developments invite different legitimate perspectives and constantly give rise to new questions and dilemmas: 1) active and early involvement of diverse stakeholders throughout the research and innovation process, 2) anticipation of alternative scenarios, including different perceptions of problems and solutions, 3) reflection on underlying values and purposes, and 4) willingness and ability to adapt responsively.

Within the GUTS program, we aim to build a reflective learning process to accompany the research program, based on Youth-Participatory Action Research (Toenders et al., 2024) and the Community of Practice approach (van Atteveldt et al., 2019). In this approach, we will bring together multiple stakeholders (e.g., GUTS researchers, youth, teachers, youth workers, policy makers) to form a community around the idea of responsible embedding of predictive methods in relation to (social) development. Through reflection and dialogue, the members of the Community of Practice will generate innovative and creative solutions and new practices regarding the responsible embedding of their research. We aim to conduct individual and focus group interviews and dialog sessions to explore the issues at stake in depth. Several Reflexive Monitoring in Action tools will be used, such as the Dynamic Learning Agenda and Eye Opener Workshops. The Dynamic Learning Agenda is a tool to explain the challenges encountered and to guide the program partners toward solving these challenges by stimulating reflection and learning. Eye Opener Workshops focus on articulating and harvesting insights on ethical issues and strategies for responsible embedding. The insights gained from the reflexive monitoring activities will directly feed into the program’s orientation and new activities, thus contributing to the responsible embedding of research in society. This has the advantage that the planned activities will lead to a better theoretical and practical understanding of the ethical issues of research and the strategies needed to develop responsiveness to these issues at the level of the individual researcher and the program team.

## 8 Conclusions

The development of self-regulatory skills and their consequences for adolescents’ contributions to society in terms of personal well-being, education, social connections, and antisocial behavior cannot be understood without considering changes in several major domains of development (Wesarg-Menzel et al., 2023). Biologically, there are marked changes in the adolescent body as it transitions from child to adult, including changes in brain structure and function (Casey, 2015). These changes manifest themselves behaviorally in the salience of affective and motivational signals and the flexible engagement of cognitive control (van Duijvenvoorde et al., 2016). Adolescence is characterized by changes that occur in the social domain, as family relationships change and adolescents take on more independent roles in society, and as peers become more important socially and romantically (Veenstra and Laninga-Wijnen, 2021). Understanding these dynamic transitions requires an examination of the bidirectional influences between social opportunities and individual brain functioning.

An urgent question of the GUTS program concerns understanding how diversity in social-economic background, peer relations and societal support structures exert an influence on how young people feel seen, heard and respected, and how they can be given opportunities to contribute to a complex world with many challenges, including climate change and inequality (Choudhury et al., 2023, Dotson and Duarte, 2020). We propose that the development of self-regulation is a key process that is influenced by and can influence the way young people navigate their social world. The central challenge to addressing these questions is how we develop valid methodologies that operationalize the social context and that we can measure the behavioral and neural signatures. We propose that with the GUTS program we recognize the mutual relationships between individual, social and societal processes on developing adolescents to understand pathways and mechanisms. To fully capture the operationalization of social context, we propose that youth-participation methods are an important source of including lived experiences of growing up in complex environments and use a responsible research and innovation approach to communicate the findings to the larger society (Green et al., 2023, van Atteveldt et al., 2019). The GUTS transdisciplinary approach with team science can provide new perspectives on connecting brain development and self-regulation in social settings to a variety of contexts.

## CRediT authorship contribution statement

**Arne Popma:** Writing – original draft, Conceptualization. **Mark de Rooij:** Writing – original draft, Conceptualization. **Lucres Jansen:** Writing – original draft, Conceptualization. **Barbara R. Braams:** Writing – original draft, Conceptualization. **Nienke van Atteveldt:** Writing – original draft, Conceptualization. **Ingmar Franken:** Writing – original draft, Conceptualization. **Gert Stulp:** Writing – original draft, Conceptualization. **Barbara Franke:** Writing – original draft, Conceptualization. **Lydia Krabbendam:** Writing – original draft, Conceptualization. **Christian Keysers:** Writing – original draft, Conceptualization. **Eveline A. Crone:** Writing – original draft, Funding acquisition, Conceptualization. **Loes Keijsers:** Writing – original draft, Conceptualization. **Thijs Bol:** Writing – original draft, Conceptualization. **René Veenstra:** Writing – original draft, Conceptualization. **Berna Güroğlu:** Writing – original draft, Conceptualization. **Anna van Duijvenvoorde:** Writing – original draft, Conceptualization. **Valeria Gazzola:** Writing – original draft, Conceptualization. **Hilleke Hulshoff Pol:** Writing – original draft, Conceptualization. **Hilde Huizenga:** Writing – original draft, Conceptualization.

## Declaration of Competing Interest

The authors have no competing interests.
