# Supplementary material for: Growing Up Together in Society (GUTS): A team science effort to predict societal trajectories in adolescence and young adulthood
Source: Dev Cogn Neurosci. 2024 Jun 6;67:101403. doi: 10.1016/j.dcn.2024.101403 (PMC11214182; doi:10.1016/j.dcn.2024.101403)
Supplement: Supplementary file 1 — Supplementary material [file mmc1.docx]

**Supplementary Information.**

**References Table 1:**

1 Adler, N. & Stewart, J. The MacArthur Scale of Subjective Social Status. (2007).

2 Frankenhuis, W. E., Roelofs, M. F. A. & de Vries, S. A. Does exposure to psychosocial adversity enhance deception detection ability? . *Evolutionary Behavioral Sciences* **12**, 218–229 (2018). <https://doi.org>: <https://doi.org/10.1037/ebs0000103>

3 Green, K. H. *et al.* Objective versus subjective socioeconomic disadvantage, experienced inequality, and wellbeing across adolescence. . *Preprint* (2024). <https://doi.org:10.31234/osf.io/mq3yt>

4 Langener, A. M., Kramer, A., Van Den Bos, W. & Huizenga, H. M. A shortened version of Raven’s standard progressive matrices for children and adolescents. . *British Journal of Developmental Psychology* **40**, 35–45 (2021). <https://doi.org:https://doi.org/10.1111/bjdp.12381>

5 Petersen, A. C., Crockett, L., Richards, M. & Boxer, A. A self-report measure of pubertal status: Reliability, validity, and initial norms. *Journal of youth and adolescence* **17**, 117-133 (1988). <https://doi.org>: <https://doi.org/10.1007/BF01537962>

6 Furman, W. & Buhrmester, D. Children's perceptions of the personal relationships in their social networks. Developmental psychology. *21*, 1016-1022 (1985). <https://doi.org:https://doi.org/10.1037/0012-1649.21.6.1016>

7 Grolnick, W. S., Ryan, R. M. & Deci, E. L. Inner resources for school achievement: Motivational mediators of children's perceptions of their parents. *Journal of Educational Psychology* **83** (1991). <https://doi.org:https://doi.org/10.1037/0022-0663.83.4.508>

8 Zarse, E. M., Neff, M. R., Yoder, R., Hulvershorn, L., Chambers, J. E. & Chambers, R. A. The adverse childhood experiences questionnaire: Two decades of research on childhood trauma as a primary cause of adult mental illness, addiction, and medical diseases. *Cogent Medicine* **6**, 1581447 (2019). <https://doi.org:https://doi.org/10.1080/2331205X.2019.1581447>

9 Kasser, T. & Ryan, R. M. Aspiration index. . *Journal of Personality and Social Psychology* (1996). <https://doi.org:https://doi.org/10.1037/t00712-000>

10 Carver, C. S. & White, T. L. Behavioral inhibition, behavioral activation, and affective responses to impending reward and punishment: the BIS/BAS Scales. . *J Pers Soc Psychol* **67**, 319–333 (1994).

11 Tangney, J. P., Baumeister, R. F. & Boone, A. L. High self‐control predicts good adjustment, less pathology, better grades, and interpersonal success,. *Journal of Personality and Social Psychology* **2**, 271–324 (2004). <https://doi.org:https://doi.org/10.1111/j.0022-3506.2004.00263.x>

12 Hawk, S. T., Keijsers, L., Branje, S. J., Graaff, J. V., Wied, M. & Meeus, W. Examining the Interpersonal Reactivity Index (IRI) among early and late adolescents and their mothers. *J Pers Assess* **95**, 96-106 (2013). <https://doi.org:10.1080/00223891.2012.696080>

13 Van Rijn, L. H. *et al.* Delay discounting in adolescence depends on who you wait for: Evidence from a functional neuroimaging study. *Submitted for publication* (2024).

14 Aron, A., Aron, E. R. & Smollan, D. Inclusion of Other in the Self Scale and the structure of interpersonal closeness. *Journal of Personality and Social Psychology* **63**, 596-612 (1992).

15 Meerkerk, G. J., Van Den Eijnden, R. J., Vermulst, A. A. & Garretsen, H. F. The compulsive internet use scale (CIUS): some psychometric properties. . *Cyberpsychology & behavior* **12**, 1-6 (2009). <https://doi.org:https://doi.org/10.1089/cpb.2008.0181>

16 van de Groep, S. & Crone, E. A. Online Prosocial Behaviors in Adolescence: Age and gender patterns for Online Emotional Support and Activism. *Preprint* (2024). <https://doi.org:https://doi.org/10.31234/osf.io/2qge7>

17 Fietze, S., Holst, E. & Tobsch, V. Germany's next top manager: Does personality explain the gender career gap? *Management Revue* **22**, 240-273 (2011).

18 Duell, N. & Steinberg, L. Differential Correlates of Positive and Negative Risk Taking in Adolescence. *J Youth Adolesc* **49**, 1162-1178 (2020). <https://doi.org:10.1007/s10964-020-01237-7>

19 Allen, J. P., Litten, R. Z., Fertig, J. B. & Babor, T. A review of research on the Alcohol Use Disorders Identification Test (AUDIT). Alcoholism: clinical and experimental research. **21**, 613-619 (1997). <https://doi.org:https://doi.org/10.1111/j.1530-0277.1997.tb03811.x>

20 van de Groep, S., Zanolie, K., Green, K. H., Sweijen, S. W. & Crone, E. A. A daily diary study on adolescents' mood, empathy, and prosocial behavior during the COVID-19 pandemic. *PLoS One* **15**, e0240349 (2020). <https://doi.org:10.1371/journal.pone.0240349>

21 te Brinke, L. W., Er Vargün, G. & Gummerum, M. Dutch adaptation of the Prosocial Behavior Questionnaire (PBQ-NL): A validity and reliability study in adolescents and early adults. . *Preprint* (2024). <https://doi.org:10.31234/osf.io/nj5sv>

22 De Koster, W., Achterberg, P., Van der Waal, J., Van Bohemen, S. & Kemmers, R. Progressiveness and the new right: The electoral relevance of culturally progressive values in the Netherlands. *West European Politics* **37**, 584-604 (2014).

23 Kasper, J., Schumacher, G. & Bakker, B. N. Establishing the Construct and Predictive Validity of Brief Measures of Affective Polarization. *Preprint* (2024). <https://doi.org:https://doi.org/10.31234/osf.io/gb32z>

24 Groeniger, J. O., Noordzij, K., Van Der Waal, J. & De Koster, W. D. C.-l. m. i. t. i. g. a. t. i. s. A. d.-i.-d. a. S. S. M. Dutch COVID-19 lockdown measures increased trust in government and trust in science: A difference-in-differences analysis. . *Social Science & Medicine* **275**, 113819 (2021). <https://doi.org:https://doi.org/10.1016/j.socscimed.2021.113819>

25 Goodman, R. Psychometric properties of the strengths and difficulties questionnaire. . *Journal of the American Academy of Child & Adolescent Psychiatry,* **40**, 1337-1345 (2001). <https://doi.org:https://doi.org/10.1097/00004583-200111000-00015>

26 Patrick, D. L., Edwards, T. C. & Topolski, T. D. Adolescent quality of life, part II: initial validation of a new instrument. Journal of adolescence. **25**, 287-300 (2002). <https://doi.org:https://doi.org/10.1006/jado.2002.0471>

27 Green, K. H., Van de Groep, S., van der Cruijsen, R., Polak, M. G. & Crone, E. A. The multidimensional wellbeing in youth scale (MWYS): Development and psychometric properties. *Personality and Individual Differences* **204**, 112038 (2023).

28 van Baardewijk, Y., Andershed, H., Stegge, H., Nilsson, K. W., Scholte, E. & Vermeiren, R. Development and tests of short versions of the youth psychopathic traits inventory and the youth psychopathic traits inventory-child version. . *European Journal of Psychological Assessment.* **26** (2010). <https://doi.org:https://doi.org/10.1027/1015-5759/a000017>

29 Cohen, S., Kamarck, T. & Mermelstein, R. A global measure of perceived stress. . *Journal of Health and Social Behavior* **24**, 386-396 (1983). <https://doi.org:https://doi.org/10.2307/2136404>
